# Current landscape of cancer genomics research in sub-Saharan Africa – a review of literature

**DOI:** 10.3389/fonc.2025.1512005

**Published:** 2025-04-17

**Authors:** Lemchukwu C. Amaeshi, Kehinde S. Okunade, Rose I. Anorlu

**Affiliations:** ^1^ Montefiore Medical Center, Department of Medicine, Bronx, NY, United States; ^2^ Oncology and Pathological Unit, Department of Obstetrics and Gynecology, College of Medicine, University of Lagos, Lagos, Nigeria; ^3^ Lagos University Teaching Hospital, Lagos, Nigeria

**Keywords:** sub-Saharan Africa, cancer genomics, genomic diversity, genomic research challenges, current initiatives, genomic research implementation

## Abstract

Cancer poses a significant public health challenge in sub-Saharan Africa, a region that has traditionally struggled with infectious diseases. Although communicable diseases remain the leading cause of mortality in sub-Saharan Africa (SSA), there has been a rise in the morbidity and mortality rates associated with non-communicable diseases (NCDs), in recent years. As of 2019, NCDs accounted for 37% of deaths, representing an increase from the 24% recorded in 2000. Cancer is fundamentally a genetic disorder, and genomic research has provided a deeper understanding of its biology leading to identification of biomarkers for early cancer detection and advancement in precision oncology. However, despite Africa’s rich genomic diversity and significant cancer burden, the continent remains underrepresented in global genomic research. This underrepresentation is mainly due to challenges such as insufficient funding, inadequate infrastructure, and a limited pool of trained professionals. However, despite these obstacles, initiatives like the H3Africa Consortium, African BioGenome Project, and Prostate Cancer Transatlantic Consortium (CaPTC), amongst others, have made significant strides in funding and developing local capacity and infrastructure for genomic research. In this review, we discuss the unique genomic characteristics of common cancers in Africa, highlight challenges faced in the implementation of genomic research, and explore potential solutions and current initiatives instituted to foster genomic research in the region.

## Introduction

The burden of cancer continues to grow exponentially. According to global cancer statistics of 2022, there were close to 20 million new cases of cancer in 2022 and almost 10 million deaths from it (1). It is anticipated that by the year 2025, the number of individuals diagnosed with cancer will reach 35 million, representing a 76.6% increase from the 2022 estimate of 20 million. In addition, it is projected that there will be 18.5 million cancer-related fatalities by 2050, which indicates an 89.7% increase from the 2022 estimate of 9.7 million (1, 2). Cancer has become an increasingly significant public health burden in sub-Saharan Africa (SSA), a region historically plagued by infectious diseases such as malaria, tuberculosis, and HIV/AIDS (3, 4). As SSA experiences epidemiological shifts, non-communicable diseases have become significant contributors to both morbidity and mortality (5). Although the precise prevalence of cancer in Sub-Saharan Africa (SSA) remains undetermined, GLOBOCAN 2022 has estimated that the five-year prevalence of cancer cases exceeds 1.7 million, including 848,311 new cancer diagnoses and 559,008 cancer-related fatalities (6). By 2030, it is projected that there will be over 1.5 million new cases and 1 million deaths (7, 8). The growing cancer burden in this region is attributed to factors such as increasing aging populations, changes in lifestyle, urbanization, and the increasing prevalence of risk factors like tobacco use, obesity, and infections (4). Inherent factors such as poverty and limited health infrastructure, including poor access to care leading to delays in diagnosis and treatment, low public awareness of cancer, and conflicting cultural beliefs, contribute to the challenges faced in cancer care in the continent (4).

Cancer genomics, a product of the human genome project completed in 2003, has revolutionized our understanding of cancer (9, 10). It has enabled a deep understanding of cancer biology by defining somatic and germline defects in individual tumors, driving carcinogenesis, and discovering targetable mutations and tumor-specific antigens. These discoveries inform the utilization and incorporation of precision oncology in day-to-day clinical practice (11).

The genomic diversity of the human population is influenced by ethnic and geographic differences, population migration, and exposure to infectious diseases. Although genetic diversity occurs in different populations, most genetic variation occurs within continents (12). Africa has the most extensive genetic and phenotypic variation among all humans, and studies have shown high nucleotide and haplotype diversity levels in Africans compared to other populations (13–15). Structural variations, like copy number variations and inversions, are higher among Africans than Europeans (16). A study by Choudhury et al. using data from the H3Africa Consortium performed a whole genome analysis of 426 individuals from 50 ethnolinguistic groups and uncovered more than 3 million previously undescribed single nucleotide variants and 62 previously unreported loci (17).

Despite Africa’s vast genetic diversity, the continent remains significantly underrepresented in human genetics research, particularly in the field of cancer genomics (16). For example, individuals of African descent account for less than 3% of participants in genome-wide association studies, predominantly from North and South Africa (17). This underrepresentation is mirrored in the publication landscape, where Africa contributes a mere 0.016% of peer-reviewed articles in cancer genomics globally (18).

For instance, Africans and people of African origin constitute less than 3% of genome-wide association studies, mainly in North and South Africa (19). Consequently, this is reflected in the number of African-authorship publications in cancer genomics worldwide, where Africa only contributes 0.016% of cancer genomics peer-reviewed publications globally (20).

Addressing the socioeconomic factors and public health concerns contributing to the cancer burden in Africa is crucial. Equally important, however, is the need for genomic research that is specifically tailored to the African population. Such research can uncover the unique factors driving carcinogenesis among Africans, enhance our understanding of cancer biology in the context of Africa’s diverse ethnic groups, and pave the way for the development of genetically tailored treatment regimens.

This review aims to examine the current landscape of cancer genomics on the continent. It will highlight the ongoing genomic research in Africa, identify challenges that impede the advancement of cancer genomics in the region and outline current strategies and initiatives designed to promote cancer genomics research in Africa.

## Unique cancer genomic profiles in sub-Saharan Africa

Breast cancer, cervical cancer, prostate cancer, colorectal cancer, and liver cancer are among the leading causes of cancer in Africa (21). Among pediatric malignancies, non-Hodgkin’s lymphoma—especially those associated with infections, such as Kaposi sarcoma and Burkitt’s lymphoma—constitute some of the most prevalent cancers in children within sub-Saharan Africa (22, 23). Rotimi and colleagues undertook a comprehensive literature review on genetic and genomic research pertaining to the African population from 1990 to 2019 (24). They identified only 375 publications centered on cancer genomics in Africa, constituting less than 0.05% of the cumulative cancer-related publications conducted in fewer than 10 of the 54 countries within the region. Within these studies, 152 genes implicated in carcinogenesis and tumorigenesis were recognized. The most extensively researched genes were BRCA1, BRCA2, TP53, EGFR, and MLH1. There exists a concern that despite the association of these genes with the most prevalent cancers in the region, malignancies such as lung cancer, ovarian cancer, and non-Hodgkin’s lymphoma—which contribute significantly to cancer mortality in the region—remain underrepresented and insufficiently investigated in genomic research throughout Africa.

We will highlight key genomic research findings related to some of these specific cancers in African populations and how they differ from other racial and geographic populations.

### Breast cancer

Breast cancer (BC) is the most common cancer in Africa (21). It continues to be a leading cause of mortality in the region, with over 185,000 new cases and over 85,000 deaths, according to GLOBOCAN 2020 statistics (25). Increased mortality is attributed to late-stage diagnosis, limited access to screening programs, diagnostic facilities, treatment options, and poverty. African women are more likely to suffer from triple-negative breast cancer (TNBC), which is an aggressive form of breast cancer. TNBC accounts for 33% of breast cancer diagnoses in Africa, compared to less than 20% in other geographical regions, with the highest incidence in East and West Africa (26–28). Genetic mutations known to increase the predisposition to breast cancer include BRCA1, BRCA2, TP53, PTEN, CDH1, ATM, CHEK2, PALB2, *BARD 1, NF1, RAD51C*, and *RAD51D* mutations​ (25–29)​. Genetic Studies conducted among women with breast cancer in countries such as Ghana, Nigeria, Uganda, Cameroon, and South Africa have found that most breast cancer cases are associated with mutations of the tumor-suppressing genes - *BRCA1 and BRCA2*. These mutations occur more frequently in these populations than in women of European, Asian, and even those of African American ancestry (29–31). A meta-analysis by Chen et al. and a Tunisian study by Mahfoudh et al. found that patients with BRCA 1 mutation are more likely to have aggressive TNBC than other germline mutations associated with breast cancer (32, 33). Other mutations, such as *PALB2 and TP53*, are also associated with significant increases in breast cancer risk in the African population (29, 31). A genomic characterization of Nigerian women with breast cancer by Pitt et al. found a higher frequency of tumor protein 53 (TP53) and GATA3 mutations in Nigerians compared to other non-Hispanic Black individuals and people from other races recruited in The Cancer Genome Atlas (34). Despite the predominance of *BRCA 1 and BRCA 2* genetic mutations among African women with breast cancer, the actual prevalence of these mutations in the general African population is unknown. It is likely that given the tumor heterogeneity of breast cancer combined with the genomic diversity of the African population, several variants of the genes responsible for breast cancer exist. However, while Genome-Wide Association Studies have been done on women of European and African American ancestry, few have been carried out solely on Africans (35). Furthermore, the full spectrum of the mutational pattern, including genetic variants of breast cancer genes beyond *BRCA 1 and 2* genes across the African population, is yet to be elucidated. A review by Mahtaab Hayat et al. on the Genetic Susceptibility of Breast Cancer in SSA populations revealed as many as 46 and more than 30 germline pathogenic variants in just the *BRCA 1 and BRCA 2 genes*, respectively (36). Population-based genomic testing would aid in determining the prevalence of other genes and their variant alleles predisposing to breast cancer. Given the high incidence and mortality of breast cancer on the continent, it is worth screening for these mutations at the primary healthcare level in patients with breast cancer and their families. However, the infrastructure and trained manpower to carry out these tests are not readily available, accessible, or affordable. Two PARP inhibitors, Olaparib and Talazoparib, have been approved by the United States Food and Drug Administration (FDA) and the European Medicines Agency (EMA) for breast cancer patients with deleterious or suspected deleterious BRCA mutations. However, these targeted therapies are neither affordable nor available for African breast cancer patients. There is a need to fully characterize the genetic profile of breast cancer in each African country and translate these results into more accessible, effective, and ultimately personalized and cost-effective therapies for African patients (37, 38).

### Cervical cancer

Cervical cancer remains a leading cause of morbidity and mortality of female cancers in sub-Saharan Africa, following breast cancer in African women, with an incidence of over 110,000 cases and over 75,000 deaths (39). The human papillomavirus (HPV) causes cervical cancer, but cancer promoters such as host genetic mutations modulate susceptibility to cervical cancer. Molecular studies on cervical cancer have identified single nucleotide polymorphisms (SNPs) and CNV associated with cervical cancer. Again, despite the burden of cervical cancer in sub-Saharan Africa, only a few genomic studies have been conducted in the African population. A GWAS study in South Africa identified genetic variants in the major histocompatibility complex regions associated with cervical cancer - *rs3129965* near *HLA-DRA*, *rs575105445* near *HLA-C*, *rs184053148* in *TRIM31*, *rs12496921* in *ROPN1B* and *rs114031308* near *TTC26* (40). The genes in these loci were implicated in antiviral immunity, DNA repair, the regulation of p53, apoptosis, cell growth, and differentiation. A literature review by Oppah Kuguyo et al. on genetic susceptibility for cervical cancer in African populations uncovered 20 studies done on genetic polymorphism from Tunisia, South Africa, Zimbabwe, Gabon, Senegal, Morocco, and Sudan (41). Out of the 30 known genes associated with cervical cancer, only nine were studied in the African population (Caspase 8, Chemokine receptor 2, Fas cell death receptor, human leukocyte antigen, Interferon-gamma, interleukin 10, *TP 53* Tumor necrosis factor-alpha, Transforming growth factor-beta 1). It was found that the effect of genetic polymorphisms such as SNPs on these genes and susceptibility to cervical cancer varies among racial and African sub-populations. For instance, the polymorphism of *CASP8 – CASP8-652 6N* ins/del in cervical cancer, which plays a role in HPV persistence, has been found in high frequencies in the African population and low in the Asian population. Despite its high frequency, it is associated with a decreased risk of cervical cancer among South African populations (42). Such discoveries demonstrate the vital contribution of common genetic variants to cervical cancer susceptibility and provide some insight into its pathogenesis in African women. HPV 16 and 18 are responsible for seventy percent of cervical cancer cases (43). However, studies have shown a higher prevalence of HPV 35 in African women with cervical cancer compared to other racial populations (44–46). Despite this association, there are few studies on the genomics and virology of HPV 35 (47). The available HPV vaccines, Cervarix and Gardasil, were developed to target HPV 16 and 18 specifically, but not HPV 35 (44). While these vaccines offer cross-protection against HPV 35, more research is needed to understand the effectiveness of these vaccines against this strain. Additionally, the genetic factors that increase susceptibility and persistence to HPV 35, as well as the factors that drive its progression to cancer, are not fully known. Again, we need more genomic research to analyze HPV 35 in the African population comprehensively, identify host genes susceptible to this strain of virus, and discover new antigens for effective multi-epitope vaccines.

### Prostate cancer

In SSA, prostate cancer is the most common cancer in men, with an incidence of over 77,000 cases (48). African men develop prostate cancer at a younger age, present with more aggressive disease, have the highest frequency of genomic risk for prostate cancer, and are more subject to tumor heterogeneity than men of European descent (49). This suggests that genetic factors unique to African populations may contribute to disease severity in the region. As in the previously discussed cancers, the African population is underrepresented in genomic research on prostate cancer. Genotypes involved in prostate cancer etiology differ across populations and ethnicities. Most of the studies on prostate cancer in Africa were conducted in South Africa, Ghana, Tunisia, and Uganda (50–52). In a GWAS, genetic variants in the *8q24, 6q22.1, and 11q13.3* loci have been recognized as strong risk factors for prostate cancer in men of African descent (53). Furthermore, men of African ancestry have a higher burden of these alleles compared to men of European or Asian descent (54). The risk of prostate cancer increased with the increased frequency of allele variants of the prostate cancer gene *CYP3A4 and SRD5A2*, which was found in higher frequencies among Africans, particularly Senegalese and Ghanaians, than other races (55). Genetic variants of the *HOXB13* gene are known to contribute to early-onset prostate cancer in African populations (56, 57). A study by The Prostate Cancer Transatlantic Consortium (CaPTC) utilizing whole exome sequencing to determine genetic variants in Nigerian patients with advanced prostate cancer identified high frequencies of mutations in the *BRCA1, BRCA2, APC, and ATM* genes. In addition, three novel prostate cancer genes were identified: *CACNA2D2, SYNE1, and ADAMTS2* (58). Although the full functions of these novel genes are yet to be characterized, they have been implicated in tumor proliferation and angiogenesis. Recent genomic studies have identified novel genetic loci that may be specific to African populations. For example, certain variants at *17q21* have been implicated in prostate cancer risk among African men, but these findings require further validation (59). The genomic variability of prostate cancer in Africa remains poorly defined, with research mainly confined to a handful of countries. Due to the genetic diversity within the African population, it is likely that numerous alleles associated with prostate cancer risk in Africans have yet to be identified. Enhanced genetic and epidemiological studies in Africa are essential to understanding the various genomic variations that play a role in the development of prostate cancer.

### Hepatocellular cancer

In SSA, hepatocellular carcinoma (HCC) is the second leading cause of cancer in men and the third in women in sub-Saharan Africa, with an estimated 38,629 incident cases and 36,592 HCC deaths in 2020 for both sexes (60). Risk factors contributing to HCC include chronic hepatitis B and C infections, alcohol abuse, aflatoxins, and metabolic dysfunction–associated steatotic liver disease (61). Several host genetic variants, such as mutations in genes encoding for several cytokines, chemokines, and human leukocyte antigens, have been found to influence the carcinogenesis of HCC (62). Although extensive research has been done on the genomics of host immune factors, few have been conducted in Africa, mainly in North and West Africa, among hepatitis C patients (62–65). One of the most studied mutations associated with HCC is the *TP53* gene mutation. Mutation in codon 249 of the *TP53* gene was identified as a contributing factor to HCC in those exposed to aflatoxins or those infected with the hepatitis virus. Studies in SSA from Nigeria, Gambia, and Senegal have shown that 36% to 66% of HCC patients have this mutation (66–69).

### Colorectal cancer

Colorectal cancer (CRC) ranks as the fifth most prevalent cancer in Africa. Despite extensive research into the genomic epidemiology of CRC, there are still limited studies on the African population. The molecular pathways associated with CRC involve microsatellite instability, chromosomal instability, and hereditary syndromes. Research shows that colorectal cancers in Africans exhibit higher rates of hereditary syndromes, earlier onset, more advanced stages, increased mucinous differentiation, and a greater prevalence of high microsatellite instability (MSI-H) compared to high-income nations (70–72). We have little information on the reason behind the high frequency of mismatch repair deficiency in sub-Saharan Africa. Studies have shown that the African population’s sporadic mutations in *KRAS, BRAF, APC, CTNNB1*, and *MLH1* contribute to MSI-H. However, the exact prevalence of these mutations is unknown, and studies on hereditary syndromes are limited to just case reports.

The dearth of genetic studies in Africa regarding the cancer burden highlights the disparity in genomic research compared to other countries, underscoring the need for large-scale genetic research to map out genetic risk factors unique to Africa that predispose individuals to carcinogenesis and ultimately to develop targeted therapies tailored for these populations.

## Challenges of cancer genomic research in sub-Saharan Africa

Despite the urgent need for continent-wide genomic research, implementing it in sub-Saharan Africa is still hindered by challenges, including infrastructure, ethical, financial, and cultural factors. These factors contribute to the global disparity in genomic research.

### Infrastructure and resources

Genomic cancer research requires dedicated laboratory centers with well-equipped infrastructure and tools for analyzing genomic data. However, many African countries have limited access to such facilities and, most of the time, have to be imported (24, 73, 74). Due to the high costs of this equipment and the bureaucracy involved in shipping, sometimes these supplies are delivered late (73). Access to advanced data generation, analysis, storage technology, and bioinformatics infrastructure to store genomic information is limited, inadequate, or absent (73). Basic amenities such as power and uninterrupted, high-speed internet connections are lacking in SSA, which also adds to the barriers to carrying out large-scale and multinational genomic research in the continent (75, 76).

### Inadequate funding

Genomic research requires significant financial investment since the equipment needed in genetic and genomic research needs to be imported, as they are not produced locally. For example, setting up a next-generation sequencing facility ranges from $100,000 to $700,000 (77). Beyond the initial purchase, importing the equipment involves substantial taxes. In addition, regular maintenance and servicing are necessary, and spare parts have to be sourced internationally. There are also expenses related to hiring and transporting skilled personnel and technicians. Many institutions, especially those in low- and middle-income countries (LMICs), struggle to secure sufficient funding for these activities. A study by Stark and colleagues revealed that since 2013, several high-income countries have invested billions of dollars in establishing national genomic medicine initiatives to promote genomic research (78). However, this does not apply to resource-limited countries, particularly in the SSA region. This finding is unsurprising, as most African countries allocate less than 1% of their GDP to medical research and development (79, 80). Moreover, although we are seeing a rising frequency of cancer cases, most African governments often focus on infectious diseases such as HIV, malaria, and TB over cancer. Researchers in these regions often rely on international grants, which are highly competitive and may come with restrictions or conditions that may be unattainable. This is heightened by inadequate government support for research infrastructure in many countries as they still struggle to strengthen their primary healthcare systems (79). The long bureaucratic process of country-specific legal and regulatory control policy sometimes leads to delayed funding from international sponsors (73). Consequently, promising research ideas may be abandoned due to lack of financial backing, and the research that does proceed may suffer from delays or compromises in quality.

Most Africans pay out of pocket for health care, and most live below the poverty line (81). The reliance on out-of-pocket payments continues to push more Africans deeper into poverty. According to WHO, over half of all impoverished people, because of out-of-pocket payments, reside in Africa. Therefore, implementing genomic research in cancer care clinical practice through cancer genetic testing becomes a challenge to the already expensive cost of cancer care. For instance, genetic testing for certain cancers ranges from $100 to $1,000 in South Africa (82). A study by Prisca et al. on the willingness of patients living with cancer to undergo genetic testing revealed that nearly one-third of participants were unwilling to pay for it. This reluctance stemmed primarily from a lack of funding, while others insisted that genetic testing should be free (83). Apart from cancer genetic testing, there is also the high cost of treating cancer patients with targeted therapy. For instance, Trastuzumab, a lifesaving therapy in patients with HER 2 receptor-positive breast cancer, averages about $36,000 per annum, making it unaffordable; very few complete a treatment course or experience treatment delays due to its high cost (84, 85). Therefore, adds to the burden of financial toxicity in Africans living with cancer.

### Shortage of skilled personnel

Genomic research is a specialized medical field requiring trained and qualified personnel, especially in bioinformatics, and advanced computational skills to manage and interpret genomic data (86). Unfortunately, in Africa, there are few trained scientists specializing in genomics. In addition, there are few training programs; universities and scientific institutions offer training in genomics, bioinformatics, and molecular biology, concentrated in South Africa (87). Often, African countries rely on skilled personnel from high-income countries, which can be capital-intensive and not sustainable. The African brain drain, which is now an epidemic, further contributes to the shortage of skilled personnel in the continent (88). According to the WHO, it is feared that Sub-Saharan Africa will be short of 5.3 million healthcare workers by 2030, and these numbers will continue to rise (89, 90).

### Ethical and cultural consideration

Sharing genomic data among researchers globally has become imperative as it engenders a broader understanding of how disease biology is affected by different ethnic and racial populations and sets the stage for developing new effective therapies in these populations. However, genomic research, particularly in human populations, raises numerous ethical and cultural challenges (91, 92). Given the relatively little genomic research in the continent, the institutional review board personnel (IRB) of most African countries may not be well experienced in reviewing protocols for genomic research, which may lead to approval delays, especially in most large-scale and multinational studies (73, 93). Data privacy is a sensitive ethical issue in genomic research. Due to limited infrastructure and expertise in sample and data analysis, African genomic data and biosamples are often stored and analyzed in international databases and biobanks. Historical experience of exploitative research among Africans, low literacy levels, and poor understanding of biobanking may deter participation in genomic research, and traditional and cultural factors affect the progress of African genomic research (73, 94). Genetic research may be viewed with suspicion or as conflicting with traditional beliefs about health and disease (95). A narrative by Mary Amoakoh-Coleman on Ethical considerations for biobanking and the use of Genomics in Africa highlighted some beliefs regarding biobanking in genomic research: 1) belief that their blood samples will be used for magic to harm or benefit others (96), 2) mistrust that blood samples will be sold to people, 3) expectation that participation in genomic research will result in immediate results and cure of their ailment, 4) the belief that biobanking, especially with blood samples will lead to depletion of the life force leading to fatigue, hospitalization with financial and economic implications (94). In a study by Ogamba et al. on Nigerian medical students’ perceptions of precision medicine, more than a third of the participants feared that genomic information may be misused by government or corporate bodies (97). These attitudes may deter individuals from participating in genomic research. Researchers must be aware of these cultural factors and tactfully engage in community dialogue to build trust and ensure the research is culturally appropriate. They must also navigate the complex legal frameworks surrounding data privacy, which vary significantly across countries and regions (91, 92).

## Promoting cancer genomics in sub-Saharan Africa

Accelerating cancer genomics in SSA involves addressing current challenges in cancer genomics on the continent and innovating strategies for advancing African genomic research globally.

### Community engagement

Community engagement (CE) is a collaborative process involving one or more groups of individuals striving towards a shared objective or common interest (98). CE plays a vital role in genetic and genomic research. It offers opportunities to educate communities about genomics and genomic research, helping to clarify concerns and misconceptions regarding the intentions of genomic research and the data and samples collected from community participants. CE also allows the research team to understand the views of the staff and local community regarding the research project. This is particularly important in the African setting, as most people have a limited understanding of genetic and genomic research, and their opinions and actions are centered around cultural beliefs. Various strategies for CE exist, either directly through community gatherings/meetings/forums or indirectly through representatives, can be employed. Still, these strategies must be flexible and tailored to the peculiarities of the community involved (99). CE should not only occur before the start of research but also be an ongoing process throughout the research and beyond. It should facilitate continuous information sharing between researchers and the community during the entire research.

To cultivate and facilitate effective CE in research, adequate funding is necessary. A qualitative study by Nankya et al. on the experiences and perspectives of key stakeholders in community engagement for genomic research in Uganda identified the lack of funding for community engagement as a limitation to its implementation (100). Funding is needed to cover transportation costs for community members, provide food and refreshments, support outreach efforts to a diverse community that may be underrepresented in research, offer financial incentives for participation in surveys, focus groups, or interviews, develop and train individuals in the field; and enable researchers to develop research tools tailored to the specific needs of the community (101). However, since most of the funds for community engagement are secured through international collaboration, there are concerns that funders may unilaterally dictate the research direction without involving the community in the design and development process. Additionally, funders might impose conditions that could undermine the values and priorities of the communities, contravening the principles of CE—collaboration, mutuality, and transparency. Therefore, funders must also receive training in the skills and knowledge of community engagement to prevent them from dominating the research process and thereby undermining the original purpose of the funding.

### Establishing centers for genomic research

To foster genomic research in SSA, more centers should be established, including genetic registries, sequencing facilities, bioinformatic tools, and biobanks. These centers should be decentralized, hopefully making genomic research more accessible and available to a broader population. No doubt, establishing these centers is capital-intensive (77). African governments and ministries of health need to prioritize funding for research and development in genomic research to promote sustainability and reduce reliance on international and private organizations. Meanwhile, looking into cost-effective tailored genomic technologies for African researchers is a considerable alternative. For example, they can consider utilizing nanopore technology as a more economical alternative to NGS or opt for targeted panels instead of whole-genome sequencing. This approach offers a more cost-effective means of studying genetic mutations, making it more feasible for resource-constrained settings. Collaboration with these organizations is still crucial in the continent’s early stages of genomic research. Regional collaboration may be helpful where multiple countries within the continent share the cost of genomic studies, infrastructure, and data analysis, thus reducing the financial burden of individual nations. For instance, in South Africa, the National Research Foundation has a budget allocated by the government for funding genomic research. The genomic facility in Kenya has helped foster genomic research in the East African region (102). Such initiatives will form the bedrock for fostering cancer genomics on the continent.

### Training and capacity building

Effectively integrating genomic research in oncology from the laboratory to clinical settings necessitates that scientists and healthcare professionals acquire the necessary skills and knowledge. Therefore, it is vital to train and empower relevant stakeholders. Despite this, various studies indicate the foundational knowledge and expertise in genomic medicine remain limited in Africa. Raising awareness about genomics should begin as early as secondary school to spark interest in genetic research among young individuals. A survey conducted by Ogamba et al. reveals a concerning finding: fewer than thirty percent of medical students grasp the fundamental concepts of genomic testing, which is troubling yet unsurprising (97). African universities should offer academic and professional training in genomics, bioinformatics, and data science. This can be enhanced through international collaborations with established institutions, sponsoring African scientists to pursue advanced training abroad, and providing incentives for them to return to bolster local research capacity, as well as instituting online training programs and workshops focused on bioinformatics and genomics, which are more cost-effective.

## Research funding

Funding is paramount in advancing cancer genomics in Africa by addressing the critical challenges facing the implementation of cancer genomics, as previously discussed. It is essential in developing infrastructure, enhancing training and capacity building, fostering multicenter research collaborations, and managing and sharing data. Moreover, funding initiatives facilitate the establishment of ethical guidelines, policies, and frameworks, ensuring that genomic research is conducted responsibly and transparently. A substantial portion of the funding for genomic research is derived from international collaboration. For instance, over the past decade, the National Institutes of Health (NIH) has awarded grants totaling up to $17 million through the H3Africa program to support genomic research, promote the training of African genomic scientists, and enhance the continent’s infrastructure (103). Other international organizations such as the African Organization for Research and Training in Cancer (AORTIC), the American Association for Cancer Research (AACR), and the Beginning Investigator Grant for Catalytic Research have played roles in the expanding cancer research in Africa, including genomic research (104, 105). African countries need to develop ways to internally generate funds for cancer research and rely less on international aid. This enables African researchers to conduct studies that address the needs and priorities of the local population, rather than conforming to the demands of international organizations. It also reduces the stress of lengthy and complex funding applications that African researchers face when seeking funds from these organizations. Locally generated funds foster successful and sustainable cancer research across the continent.

One of the most effective ways to generate local funding is through increased government investment. African governments should dedicate more of the national health budget, specifically to cancer research. This commitment would not only demonstrate the political will to address the cancer burden but also help establish sustainable funding streams for cancer research. Local philanthropy and community-based funding initiatives can also make a significant difference. Engaging high-net-worth individuals and local philanthropists to support cancer research can create a reliable funding base. Establishing cancer research endowment funds, where contributions accrue interest over time, can ensure long-term sustainability. Community-driven fundraising efforts, such as charity runs and online crowdfunding campaigns, can mobilize public support while raising awareness about cancer research.

## Genomic research initiatives in sub-Saharan Africa

Several initiatives are actively promoting genomic research in Africa. These efforts aim to enhance health outcomes, understand genetic diversity, and rectify the underrepresentation of African populations in worldwide genomic data. These efforts emphasize disease prevention, precision medicine, and strengthening the capacities of African researchers. Herein, we highlight a few of these key initiatives.

### The human heredity and health in Africa consortium

Established over a decade ago, the H3Africa consortium is recognized as one of Africa’s most prominent and influential programs supporting population-based genomic research (106). The program has created the necessary knowledge, infrastructure, and database to empower African scientists to conduct genomic studies and aid healthcare professionals in understanding the genomic factors of prevalent diseases, notably cancer. Supported by the National Institutes of Health (NIH) and the UK Wellcome Trust, H3Africa has launched various cancer genomics projects targeting breast, cervical, and prostate cancers, which significantly impact African communities. Its initiatives include the establishment of H3ABioNet, a database providing access to genetic information from diverse samples, and the H3 Biorepository Program, which oversees bio samples from repositories in Uganda, Nigeria, and South Africa. Additionally, it has initiated studies focusing on the ethical and social dimensions of genomic research, tackling critical issues like consent, community involvement in research, and participant feedback. Through these capacity-building initiatives, H3Africa has greatly improved local genomic research resources and expertise, allowing African scientists to spearhead cancer genomics research across the continent. H3Africa consortium has facilitated cross-border collaborations and ensured that genomic data from diverse populations are shared and utilized effectively (107–109). These collaborations help overcome some of the limitations in infrastructure and capacity by providing access to advanced sequencing technologies, bioinformatics expertise, and computational resources that may not be available locally.

### African BioGenome project

This is a collaborative initiative across Africa aimed at improving the generation, analysis, and application of genomic data to promote sustainable biodiversity on the continent (80). In 2023, it initiated the Open Institute for Genomics and Bioinformatics Regional Workshops, conducting 28 workshops on biodiversity genomics and bioinformatics in 11 African nations (110, 111). These workshops have engaged over 3,700 registered participants, with 408 scientists receiving training in molecular biology, genomics, and bioinformatics, as well as the ethical, legal, and social aspects concerning the acquisition of genetic resources. The initiative has encouraged conversations with strategic leaders about their roles in building institutional infrastructure. The Open Institute aims to utilize genomics to improve and preserve African biodiversity.

### African genome variation project

This initiative offers essential resources for designing and analyzing genomic studies targeting subpopulations in Sub-Saharan Africa (SSA). It operates within a comprehensive, collaborative network formed by scientists from the African Partnership for Chronic Disease Research (112). The project aims to genotype 2.5 million genetic variants across ten ethnic groups, yielding profound insights into genetic and genomic diversity among African populations and ethnolinguistic groups. So far, it has gathered genotypes from 1,481 individuals belonging to 18 ethnolinguistic groups, alongside whole-genome sequences from 320 individuals throughout SSA (113). The initiative has evolved to provide insights into the unique genetic diversity of African populations, making it a valuable resource for global genomics research, particularly for understanding complex diseases like cancer.

### African genomic medicine training initiative

AGMT was established in 2016 during a conference organized by the African Society for Human Genetics and the H3 Africa Consortium in Senegal. It represents the first large-scale community-based training initiative implemented throughout Africa (114). This initiative emerged from the necessity to address training requirements and to create a foundational framework for genomic medicine within Africa by leveraging expertise from various regions of the continent to develop a comprehensive training program tailored for African healthcare professionals, which can subsequently be adapted to meet the diverse needs of different African nations. The strategies instituted to address these requirements include (1) the development of short courses into diploma-level content as well as graduate and post-graduate programs aimed at healthcare and research professionals; (2) training for patients who aspire to become advocates within the genetics and genomics field; and (3) public education in genomic medicine.

### African cancer genome consortium

One of the most notable cancer genomic research initiatives is ACGC, a collaborative network of researchers and institutions across Africa and beyond, focused on generating a comprehensive genomic understanding of cancers affecting African populations. The ACGC aims to map out cancer-associated genetic mutations, identify biomarkers for early detection, and develop region-specific therapeutic approaches. The consortium collaborates with leading international institutions such as Sylvester Comprehensive Cancer Center at the University of Miami Miller School of Medicine and Fox Chase Cancer Center in Philadelphia, ensuring access to state-of-the-art genomic technologies and expertise.

### Prostate cancer transatlantic consortium

CAPTC is a collaborative network established in 2005, comprising prostate cancer scientists, clinicians, survivors, and advocates from North America, Europe, the Caribbean Islands, and Africa. The consortium aims to address the disproportionate burden of prostate cancer among men of African ancestry globally by exploring genetic and environmental factors to develop targeted approaches for eliminating disparities. As discussed earlier, CaPTC recently conducted whole-exome sequencing of 45 Nigerian patients with advanced-stage treatment-naïve prostate cancers, identifying mutations in DNA repair genes, in addition to the discovery of novel cancer associated genes in the Nigerian population (58). This research underscores the importance of including diverse populations in genomic studies to develop tailored interventions. Furthermore, it seeks to develop novel, culturally sensitive tools for studying the social and behavioral determinants of prostate cancer disparities among men of African ancestry globally. It also seeks to provide cancer researchers from underrepresented regions with the resources, mentorship, and opportunities to contribute to cutting-edge research in prostate cancer.

## Impact of cancer genomics research on clinical practice in Sub-Saharan Africa

Cancer genomics research has revolutionized the field of oncology, leading to advancements in diagnostics and personalized medicine (11, 115). In translating cancer genomic findings into clinical practice, clinical oncologists need access to genetic tests, sequencing technologies, data analysis infrastructure, and decision support tools to make informed clinical assessments when treating cancer patients. As mentioned earlier, while these tools may be available to oncologists in high-income countries, they are limited in low- and middle-income regions such as Africa. However, although not on par with high-income countries, Sub-Saharan Africa is increasingly expanding genomic research, integrating it into its cancer research programs, and implementing genomic medicine in clinical practice (4, 116). Countries such as South Africa, Nigeria, Kenya, and Ghana have significantly contributed to genomic research in Africa. It is hoped that other African countries will follow in the footsteps of these countries.

Genetic testing for mutations such as BRCA1 and BRCA2, commonly associated with breast and ovarian cancer, is increasingly being performed using cost-effective means for early detection and risk assessment in SSA (117–119). For instance, in South Africa, genetic testing for the BRCA1 and BRCA2 genes in breast cancer has been implemented at the primary healthcare level for over two decades, with plans to upscale to a multigene next-generation sequencing (NGS) panel (120). Genomic research in cervical cancer has enabled the development of HPV vaccines against the high-risk HPV subtypes- 16 and 18. Three vaccines have dominated the market – Gardasil, Gardasil9 and Cervarix. In 2011, Rwanda was the first African country to incorporate HPV vaccination into its national immunization program. As of 2023, 29 out of the 54 countries now have HPV vaccination in their immunization program (121). Although precision medicine is still in its infancy in SSA, targeted therapies, such as tyrosine kinase inhibitors, through international collaboration are becoming accessible in Africa and have remarkably reduced the disease burden of certain cancers like chronic myeloid leukemia. Partnerships with pharmaceutical companies and global health organizations have facilitated access to some therapies at reduced costs. For instance, through the Glivec International Patient Assistance Program, many patients in low and middle-income countries with chronic myeloid leukemia can receive Glivec (imatinib) at no cost (122).

In the future, personalized treatment plans designed for the genetic diversity of African populations will enhance treatment efficacy and minimize adverse effects. Nevertheless, incorporating genomic research and medicine into current healthcare systems demands governmental support and genuine international collaboration.

## Conclusions

The rising burden of cancer within Sub-Saharan Africa, along with its distinctive genetic diversity, underscores the critical need to advocate for the incorporation of cancer genomics into clinical practice. However, achieving this goal requires sustained investment in infrastructure, capacity building, and policy endorsement. Considerable progress has been achieved in promoting cancer genomics research in Sub-Saharan Africa through initiatives such as H3Africa, the African BioGenome Project, the African Cancer Genome Consortium, the African Organization for Research and Training in Cancer, and the Prostate Cancer Transatlantic Consortium. Efforts in cancer genomic research across Africa have led to the genomic profiling of prevalent cancers, which has identified common mutations characteristic of the region and are now being implemented in clinical practice. Genetic testing for commonly occurring oncogenes associated with breast cancer—specifically BRCA1 and BRCA2—is progressively becoming a standard procedure in numerous African countries for patients diagnosed with breast cancer. There is increasing advocacy for the administration of the HPV vaccine targeted at young women, which is being integrated into immunization programs across various African nations, and efforts to develop HPV vaccines to include HPV subtypes unique to Africa. As genomic research and its applications continue to grow in Africa and become integrated into standard oncology practice, there is hope of reversing the current epidemiological trajectory of cancer on the continent and providing new region-specific strategies for the global conquest of cancer.
